# Past experience shapes ongoing neural patterns for language

**DOI:** 10.1038/ncomms10073

**Published:** 2015-12-01

**Authors:** Lara J. Pierce, Jen-Kai Chen, Audrey Delcenserie, Fred Genesee, Denise Klein

**Affiliations:** 1 Department of Psychology, McGill University, 1205 Avenue Dr Penfield, Montreal, Québec, Canada H3A 1B1; 2 Centre for Research on Brain, Language, and Music, Rabinovitch House, McGill University, 3640 Rue de la Montagne, Montreal, Québec, Canada H3G 2A8; 3 Neuropsychology/Cognitive Neuroscience Unit, McGill University, Montreal Neurological Institute, 3801 Rue University, Montreal, Québec, Canada H3A 2B4

## Abstract

Early experiences may establish a foundation for later learning, however, influences of early language experience on later neural processing are unknown. We investigated whether maintenance of neural templates from early language experience influences subsequent language processing. Using fMRI, we scanned the following three groups performing a French phonological working memory (PWM) task: (1) monolingual French children; (2) children adopted from China before age 3 who discontinued Chinese and spoke only French; (3) Chinese-speaking children who learned French as a second language but maintained Chinese. Although all groups perform this task equally well, brain activation differs. French monolinguals activate typical PWM brain regions, while both Chinese-exposed groups also activate regions implicated in cognitive control, even the adoptees who were monolingual French speakers at testing. Early exposure to a language, and/or delayed exposure to a subsequent language, continues to influence the neural processing of subsequently learned language sounds years later even in highly proficient, early-exposed users.

## Abstract (web summary)

Whether brief early exposure to a language affects future language processing is unclear. Here Pierce *et al.* show that brain activity evoked by French pseudowords in monolingual French speaking Chinese adoptees is different from French children with no exposure to Chinese and similar to bilingual Chinese children.

## Graphical abstract

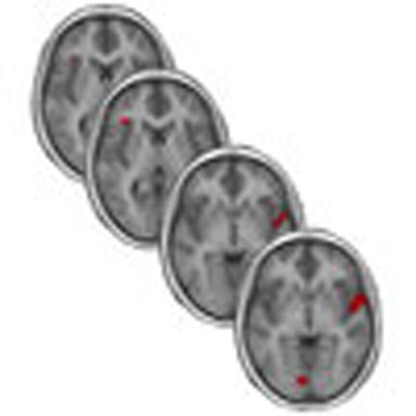

The impact of early developmental experiences on later neural outcomes is a compelling question. It is a particularly relevant issue in the domain of language learning given the wide variety of linguistic experiences children encounter in an increasingly global world. It has long been suggested that the most rapid pace of learning takes place during the first years of life and that during this time of heightened neuroplasticity the brain is optimally predisposed to collect and store basic information about the world (for example, simple visual elements and basic units of sound). Hearing a language during this time tunes infants' brains to the sounds of that language, and neural representations of these sounds are established^1^,^2^,^3^. These representations, in turn, are thought to act as a foundation for the acquisition of progressively more complex and hierarchically organized information about that language, such as increasingly complex vocabulary and grammar^4^. Importantly, this implies an ongoing relationship between early-established neural representations and more complex, higher level abilities that are acquired years or even decades later. The adaptive value of this relationship is clear when environmental contexts remain the same or similar because it allows the developing organism to build more complex language abilities on this early neural foundation. However, we have little empirical evidence if, in fact, and how these early experiences impact later neural processing. In a recent publication^5^, we showed evidence for the maintenance of neural templates for the processing of Chinese sounds in a group of international adoptees whose exposure to their birth language (Chinese) was totally discontinued when they were 12.8 months of age, on average. From that point on they were exposed to and spoke only French and had no conscious recollection of their birth language when tested more than a decade later. However, when these participants listened to a linguistic element present only in Chinese, and not their current language (French), their pattern of brain activation precisely matched that observed in native Chinese speakers who had spoken Chinese continuously since birth and had acquired French as a second language at approximately the same age as the adoptees. Importantly, the pattern of activation demonstrated by the adoptees differed significantly from the monolingual French speakers who had never been exposed to Chinese, despite the fact that all participants heard identical acoustic stimuli. The fact that the neuro-cognitive responses of the adopted participants closely matched those of the native Chinese speakers provides evidence that the neural representations supporting the processing of that language had been acquired during the first months of life and were not overwritten or lost overtime but maintained in the brain. In the present study, we sought to examine if these early-acquired language representations influence the neuro-cognitive processing of a second language.

More specifically, in the present study, we investigated neuro-cognitive processing during engagement in a phonological working memory (PWM) task in French in these same three groups. PWM is a component of executive functioning responsible for storing and manipulating incoming speech sounds in memory^6^. PWM processes use language-specific speech sounds as one mechanism for facilitating the acquisition and processing of vocabulary and grammar in that language^7^. Of particular relevance to the present study, PWM processes may rely on language-learning experiences that occur during the earliest stages of language acquisition when infants' brains become fine-tuned to the specific phonetic units of their native language^8^,^9^,^10^. However, although there is some behavioural evidence suggesting that PWM is sensitive to experiences that occur during these earliest stages^11^,^12^, this has not been demonstrated at the neural level.

PWM or verbal working memory processes have been differentiated from other types of working memory, such as visual and spatial, based on the brain regions that are activated. For example, greater activation has been observed for verbal as opposed to non-verbal memory tasks in left frontal and temporal lobes^13^, including left inferior frontal gyrus (BA 44/45) (ref. 14). Similarly, patients with left, but not right, insula lesions have shown deficits on verbal, but not spatial, working memory tasks^15^. Indeed, left inferior frontal and insular regions are thought to be important for responding to phonetic details^16^,^17^,^18^,^19^,^20^, are strongly connected with other classic language processing regions^21^,^22^, and have been found to be more active during PWM processing in ‘good' as opposed to ‘poor' language learners^23^. In contrast, while there is some overlap in neural activation between verbal and non-verbal memory processes, non-verbal memory tasks have been shown to elicit greater activation than verbal memory tasks in frontal regions, such as bilateral posterior superior frontal gyrus and left posterior medial frontal cortex^14^. Thus, the neural system thought to underlie phonological, as opposed to other types of, working memory, appears to consist primarily of a fronto-parietal network including the left inferior frontal cortex and left anterior insula (BA 44/45/13), left supramarginal gyrus and inferior parietal lobule (BA 40)^17^,^18^,^24^,^25^ and left and right superior temporal gyri (BA 22/42)^14^. While these brain regions have also been implicated in other language processes, the present discussion is limited to their role in PWM processing.

Importantly, recruitment of these or other regions during PWM processing may depend on language experience. For example, it has been found that individuals who speak more than one language demonstrate increased activation during PWM tasks in bilateral middle frontal gyri, bilateral superior temporal gyri and bilateral inferior parietal lobule as memory load increases^23^. According to Chee *et al.*^23^, activation in these regions indicates general attention and goal directed rather than language-specific processing, and these regions have also been found to be involved in cognitive control processes^26^. Thus, individuals who speak more than one language may recruit certain ‘non-language' regions during the performance of PWM tasks^26^,^27^, in addition to the brain regions typically observed in monolinguals. This may provide the basis for some of the cognitive advantages observed in bilingual speakers^28^.

To investigate how early language experiences might influence the neural processing of language sounds, we examined three groups of highly proficient French speakers (children and adolescents who ranged in age from 10–17 at the time of testing). While all groups had been exposed to French and used French daily from very early in life, the earliest language-learning experiences of each group differed. One group had been exposed only to French from birth (monolingual French participants) and had no experience with any other language. Another group was exposed to Chinese from birth, began learning French before age three, and maintained both languages at the time of testing (Chinese–French bilinguals). This group is a case of early delayed exposure to a second language (French) along with continued exposure to and use of the birth language. The third group was comprized of internationally adopted (IA) children from China. They had been exposed to Chinese from birth and began learning French at the time of adoption, also before age three; however, they abruptly discontinued exposure to their birth language (Chinese) at the time of adoption. The IA group experienced the same delay as the bilingual group before French onset, but is of particular interest since they had had exclusive exposure to French since the time of adoption (between 6 and 25 months of age), with no exposure to Chinese. Because of this unique situation, we were able to examine the influence of early, but discontinued, language experience on the neural processing of a later acquired language (French). Because the adopted and bilingual participants acquired their second language so early in life, during a period of heightened neuroplasticity for language learning, one might expect their second-language processing to resemble that of first language learners (see ref. 29 for a discussion). However, it could also be the case that neural commitment to the phonological properties of the native language^1^,^3^,^9^ would change the way second-language representations are encoded and/or subsequently processed. If this were the case, this would indicate that even the earliest language learning experiences exert a lasting effect on the neural processing of subsequently acquired languages.

Using functional magnetic resonance imaging (fMRI), we recorded brain activation while these three groups of participants performed a PWM task (n-back task) with French pseudo-words. Performance on such a task requires PWM since participants must retain stimuli in short-term memory to successfully complete the task^23^. Pseudo-words, or non-words, are thought to provide the best assessment of PWM because they capture responses to the sounds of a language without interference from other learned aspects of a language, such as meaning and grammar^6^. Participants were instructed to respond with a button press when they heard a target in a sequence of pseudo-words. In the 0-back condition, the target corresponded to the first pseudo-word presented; in the 1-back condition, the target occurred each time a pseudo-word matched the immediately preceding pseudo-word; and in the 2-back condition the target occurred when a pseudo-word matched that presented two positions prior. We show that, even in highly competent French speakers, differences in early experience with that language resulted in activation patterns that deviated from monolingual speakers who had been exposed to that language from birth. This was true even for participants who discontinued their birth language and became monolingual speakers of their second language, French, demonstrating the unique and lasting influence of early language experience on later brain organization. These results provide neural evidence that language experiences at birth, and/or relatively short delays in exposure to subsequent languages, might influence the processing of later learned languages even in highly proficient second-language speakers.

## Results

### Behavioural analysis

There were no behavioural differences in the groups on performance measures assessed both inside and outside of the scanner. Accuracy and reaction time were compared using 3 × 3 analysis of variances (ANOVAs) with group (monolingual, bilingual, IA) and task (0-back, 1-back, 2-back) as factors. In terms of accuracy, there was a significant main effect of task (*F*(2, 64)=5.54, *P*=0.006). *Post hoc* paired *t*-tests (*n*=35) revealed that, as expected, participants were significantly more accurate on the 0-back task (*t*(1, 34)=4.04, *P*=0.000), and the 1-back task (*t*(1, 34)=2.09, *P*=0.044) than on the 2-back task. There was no significant difference in accuracy between the 0-back and 1-back tasks (*t*(1, 34)=1.04, *P*=0.306; mean accuracy 0-back: 94.2%, 1-back: 93.0%, 2-back: 90.1%). Importantly there was no main effect of group (*F*(2, 32)=1.41, *P*=0.258); nor was there a significant interaction (*F*(4, 64)=0.968, *P*=0.432), indicating that accuracy did not differ between groups (mean accuracy monolingual: 90%, bilingual: 92%, IA: 94%).

In terms of reaction time, there was a significant main effect of task (*F*(2, 64)=19.65, *P*=0.000). *Post hoc* paired *t*-tests (*n*=35) revealed that performance on the 0-back task was significantly slower than the 1-back task (*t*(1, 34)=2.83, *P*=0.008), but faster than the 2-back task (*t*(1, 34)=−3.05, *P*=0.004). Reaction time on the 1-back task was significantly faster than the 2-back task (*t*(1, 34)=−7.22, *P*=0.000; mean reaction time 0-back: 1,047.25 ms, 1-back: 976.67 ms, 2-back: 1,115.25 ms). Faster performance on the 1-back compared with the 0-back task may reflect the fact that targets in the 1-back condition immediately followed their cue. Because the cue was the most recent stimulus presented it may have primed participants, leading them to respond faster when they heard the subsequent target. However, this should not affect our overall interpretation of the results. Again there was no main effect of group (*F*(2, 32)=0.796, *P*=0.460); nor was the interaction significant (*F*(4, 64)=0.844, *P*=0.502), indicating that reaction times in different conditions did not differ between groups (mean reaction time monolingual: 1,078 ms, bilingual: 1,036 ms, IA: 1,034 ms).

One-way between groups ANOVAs (monolingual; *n*=10, bilingual; *n*=11, IA; *n*=21) also indicated no significant differences between the groups on a battery of tasks conducted outside the scanner, including the Wechsler block design subtest—a test of spatial memory (monolingual=8.3, bilingual=8.3, IA=8.9, *F*(2, 40)=0.401, *P*=0.672), or on sentence recall (monolingual=7.2, bilingual=6.1, IA=6.7, *F*(2, 40)=0.526, *P*=0.595), or non-word repetition, both measures of verbal short-term memory (monolingual=37.9, bilingual=37.5, IA=37.5, *F*(2, 40)=0.196, *P*=0.822). There were also no significant differences between groups on a test of receptive vocabulary (monolingual=114.7, bilingual=111.7, IA=101.7, *F*(2, 40)=0.699, *P*=0.503). However, there was a significant difference between groups on a test of expressive vocabulary (monolingual=95.6, bilingual=91.3, IA=107.9, *F*(2, 40)=6.385, *P*=0.004). *Post hoc t*-tests revealed that the IA participants scored higher than both bilinguals and monolinguals on this test (*P*<0.05 for both comparisons), and there were no significant differences between these latter two groups. Thus, irrespective of their early language experiences, all groups displayed high proficiency in French.

### fMRI analysis

To investigate whether patterns of activation differed between the groups during PWM processing, a 3 × 3 ANOVA was performed with group (French monolingual, Chinese–French bilingual, IA) and condition (0-back, 1-back, 2-back) as factors, and age and duration of exposure to French as covariates. Results revealed a significant main effect of group in the left inferior frontal gyrus and anterior insula, inferior temporal gyrus, inferior parietal lobule, and cingulate gyrus, in the right pre-central gyrus, middle temporal gyrus, superior parietal lobule, and precuneus, and in bilateral superior temporal gyri (STG) (Table 1). There was a significant main effect of condition in the left superior frontal gyrus, inferior parietal lobule and posterior cingulate, and in the right anterior insula, anterior cingulate and middle frontal gyrus (Table 1). The group by condition interaction was not significant.

**Figure 1:**
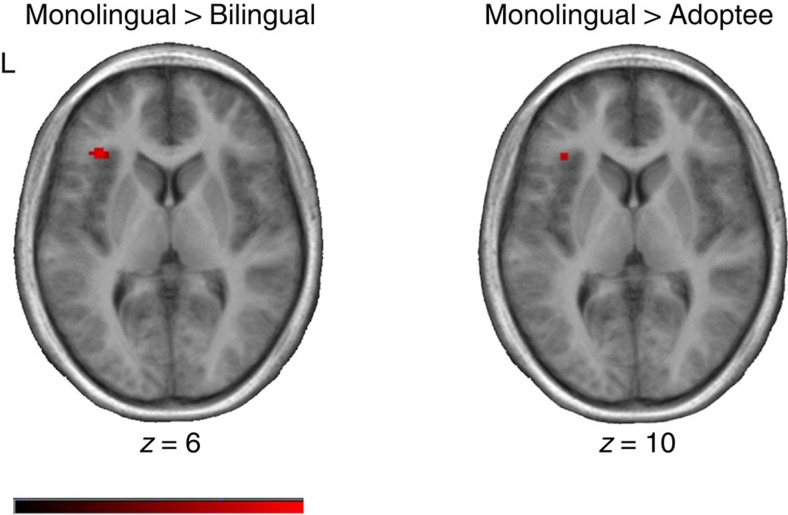
Brain regions showing increased activity in monolinguals compared with IA and bilingual groups. t-maps showing activation patterns for between-group subtractions of monolinguals >bilinguals; and monolinguals >international adoptees, collapsed across conditions and overlaid on the average anatomical t1-weighted images of each group. Slices are shown in the axial plane and are taken from the coordinate displaying the highest t-value for each group in this subtraction. The colour scale codes the range of t-values for the data (ranging from 0 to 8), and the left hemisphere is on the left side in all horizontal sections. Monolinguals activated left insula to a greater degree than the other groups. Monolinguals *n*=10; bilinguals *n*=12; international adoptees *n*=21.

**Figure 2:**
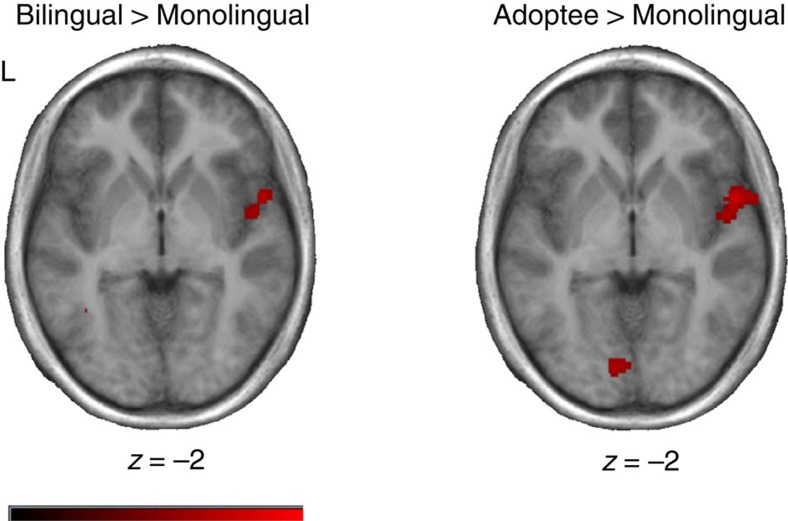
Brain regions showing increased activity in bilingual and IA groups compared to monolinguals. t-maps showing activation patterns for between-group subtractions of bilinguals >monolinguals; and international adoptees >monolinguals, collapsed across conditions and overlaid on the average anatomical t1-weighted images of each group. Slices are shown in the axial plane and are taken from the coordinate displaying the highest *t*-value for each group in this subtraction. The colour scale codes the range of *t*-values for the data (ranging from 0 to 8), and the left hemisphere is on the left side in all horizontal sections. Visible in this image is the activity in the right temporal region where bilingual (left) and IA participants (right) show significantly greater activation than monolinguals. Monolinguals *n*=10; bilinguals *n*=12; international adoptees *n*=21.

Between-group comparisons were carried out to investigate the nature of the group main effect. French monolinguals showed significantly greater activation than both the Chinese–French bilingual and IA participants in a cluster encompassing left inferior frontal gyrus and anterior insula and in right middle temporal gyrus (Table 2; Fig. 1). They also showed greater activation than the bilinguals in the right anterior insula and middle frontal gyrus, and greater activation than IA participants in left frontal pole, left inferior parietal lobule and right superior parietal lobule (Table 2). In contrast, both the Chinese–French bilinguals and IA participants activated left cingulate gyrus, right precuneus and bilateral temporal gyri (right >left) more strongly than the French monolinguals (Table 2; Fig. 2); the IA participants additionally activated the right pre-central gyrus (see Table 2).

**Figure 3:**
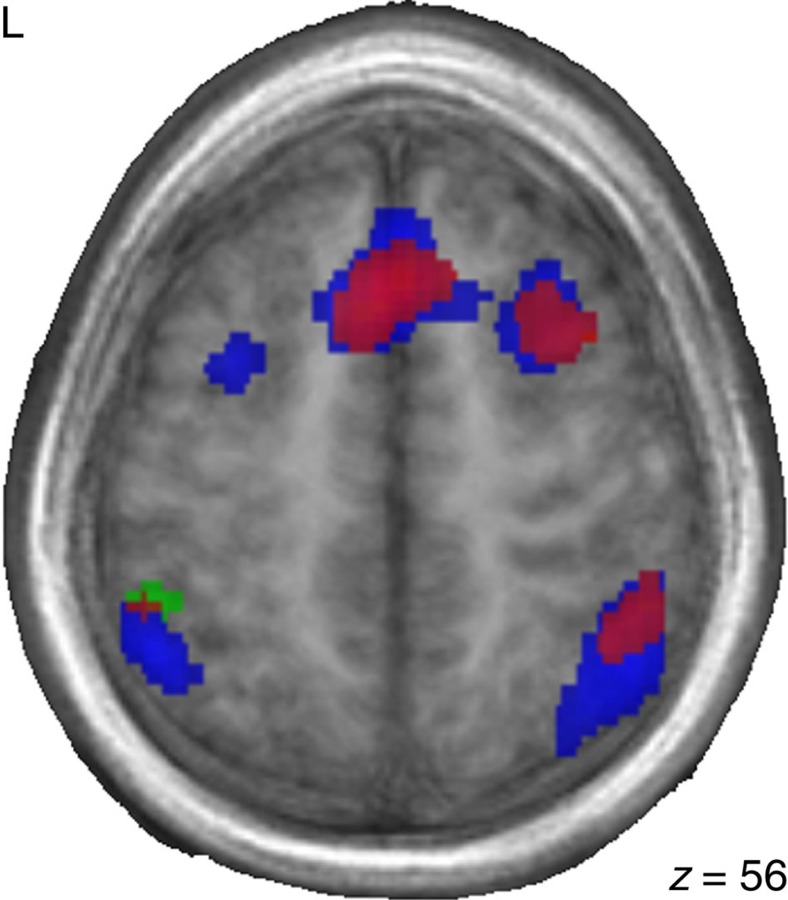
Similarity in activation patterns between bilingual and IA groups. t-map showing overlay of activation for bilinguals (red), international adoptees (blue), and monolinguals (green) during the 2-back condition. Slices are shown in the axial plane and demonstrate the similarity between the bilingual and internationally adopted groups. The left hemisphere is on the left side. Monolinguals *n*=10; bilinguals *n*=12; international adoptees *n*=21.

To investigate the nature of the condition main effect, each level of the PWM task (0-back, 1-back, 2-back) was compared (Table 3). Collapsed across groups, the 2-back (most difficult) as compared with the 0-back (easiest) condition revealed significantly greater activity in the left superior frontal gyrus and inferior parietal lobule, and the right anterior insula and middle frontal gyrus. The 2-back condition compared to the 1-back condition activated the right anterior insula and middle frontal gyrus. The 0-back condition elicited greater activation than the 2-back condition in the right subcallosal gyrus and posterior cingulate. The 1-back condition also elicited greater activation than the 2-back condition in the right subcallosal gyrus. No other comparisons were significant. Peak coordinates for each group at each condition are presented in Table 4. Visible in Fig. 3 is the close overlap between the bilingual and IA participants, which differs from French monolinguals.

Because there were significant differences between the 2-back and 0-back conditions and because we were interested in regions that increased activation as a result of memory load based on previous research^23^, we examined activation of the 2-back (most difficult) minus 0-back (easiest) conditions for each group. Strikingly, again, the results indicated a different pattern between the groups with and without delayed exposure to French. Specifically, the French monolingual group showed no significant peaks in response to increasing memory load. In contrast, both the Chinese–French bilingual and IA groups significantly activated a large cluster in the right middle frontal gyrus and inferior parietal lobule. IA participants additionally activated the left inferior parietal lobule and the right caudate and globus pallidus (Table 5). The fact that the difference between these groups and the French monolinguals was so robust is noteworthy given the former's very short delays in onset of exposure to French acquisition—all had intensive exposure to French beginning between 6 and 36 months of age.

Because of the difference observed between the French monolinguals and both other groups in left anterior insula, we examined whether this region indeed functioned as part of the PWM network for the French monolingual group and, at the same time, whether functional connectivity patterns differed across groups. To do this, we used a psychophysiological interactions (PPI) analysis. In this analysis, the left insula was used as a seed region (see details in Method) to identify, across the whole brain, other regions whose co-activation with left insula increased during task performance, implying a functional relationship^30^.

For the French monolinguals, the left insula was functionally connected with several regions typically associated with the PWM system^17^. In the left hemisphere, these included the middle temporal gyrus, pre- and post-central gyri, supramarginal and angular gyri, and inferior parietal lobule. It was also connected with the frontal pole on the right (Table 6). In contrast, for the Chinese–French bilinguals and IA participants, the left insula region was not functionally connected to these left hemisphere brain areas. In the bilingual group the left insula was not significantly connected to any regions, and in the IA group it was functionally connected only to the right frontal pole and right middle frontal gyrus.

To investigate whether timing and duration of exposure to French were related to the distinct activation patterns observed in the bilingual and IA groups, we collapsed the results from these groups and performed a whole brain, voxel-wise linear regression. Predictor variables included in this analysis were age of acquisition (AoA) of French and duration of exposure to French (current age minus AoA). We first wanted to see whether AoA was significantly related to activation in any brain area during task performance (that is, within the 2-back condition against a silent baseline) or as an effect of memory load (that is, within the 2-back condition against the 0-back condition). We found that activation was not related to AoA in any brain area for any condition.

We then examined whether participants' duration of exposure to French was significantly related to their brain activation patterns. Indeed, duration of French exposure was positively related to activation in several clusters. In the 2-back minus baseline subtraction, significant peaks were observed in the left hemisphere, including the superior frontal gyrus, lateral occipital cortex and cerebellum. duration of exposure to French was also positively correlated with activation of the supramarginal/angular gyrus and the caudate bilaterally; and in the right hemisphere with the frontal orbital cortex, frontal pole, middle frontal gyrus and middle temporal gyrus. In the 2-back minus 0-back subtraction, significant clusters were observed in right caudate, right middle frontal gyrus and right supramarginal/angular gyrus (Table 7). Thus, irrespective of the comparison, the longer the participants with delayed exposure to French had been speaking French, the more they recruited regions associated with both attention/cognitive control (for example, caudate and superior/middle frontal gyrus) and working memory processes (for example supramarginal/angular gyri). While not solely implicated as cognitive control regions, supramarginal/angular gyri have been implicated as part of a cognitive control network in bilingual speakers^31^.

To ensure that any effects were not due to maturational changes, given the wide age range tested, we conducted a whole brain, voxel-wise linear regression with current age as a predictor. Current age was not significantly associated with activation in any brain area for any condition.

## Discussion

It has long been suggested that early language learning experience impacts later development^1^, but what is not known is how this is related to underlying neural organization. Here we show, strikingly, that even relatively short delays in exposure to French, and/or early exposure to another language, lead to different neural patterns for processing the sounds of that language than is found in native speakers. Although our three learner groups were all highly proficient speakers of French and had had many years of experience with that language, the differences in language learning that they experienced within their first 3 years of life appear to have affected their patterns of brain activation years later as assessed by a PWM task in French. Crucially, this occurred even in cases where the birth language had been discontinued and participants had become monolingual speakers of their second language, French, underscoring the unique contribution of early experiences on neural processing of a later learned language.

It would appear, in our participants, that early exposure to Chinese and/or delayed exposure to French affected the brain activation patterns that are typically involved in working memory processing, including brain areas that are more strongly activated by verbal stimuli or language sounds^17^, as well as regions more typically associated with non-verbal working memory and those involved in more general attention and cognitive control^14^,^23^. In relation to the former, highly proficient French-speaking IA children and Chinese–French bilinguals, who experienced very short delays in exposure to French, recruited left inferior frontal gyrus and anterior insula more weakly when processing French phonological units in unfamiliar French-like stimuli than monolingual French speakers. While the insular region has been implicated as a key area in a variety of general cognitive functions and is a hub in proposed salience, central executive and default mode networks^32^,^33^, the left anterior insula has also been found to be active during verbal or PWM processing in a way that differs from these more general mechanisms. For example, in response to verbal memory tasks the left, in contrast to the right, anterior insula is more strongly activated^14^,^15^, and this typically occurs along with several ‘classic' frontal, temporal and parietal language regions with which the left insula shares functional and structural connections^21^,^22^. That the French monolinguals in the present study showed greater activation than the other groups in the left anterior insula, that this activation extended into inferior frontal gyrus in the left hemisphere only, and that functional connectivity was observed between left insula and other left-lateralized regions typically implicated in the PWM network, together suggest more ‘language-specific' processing of these French sounds by this group who was exposed to French from birth. Concomitantly, weaker recruitment of this region in the IA and bilingual participants may reflect the processing of these French sounds within a neural system that was initially set-up to process a different language. Of note, such findings do not mean that left insula cannot be recruited when processing non-native phonology. Indeed, other studies have reported such activation^23^,^34^,^35^,^36^, and all groups in the present study demonstrated left insular activation to some degree. However, the present results indicate that the insula may not be recruited in the same way or to the same extent as it is during native language processing.

In contrast to the typical PWM activation pattern observed in the French monolingual participants, the bilingual and IA participants more strongly activated several areas that have been implicated in non-verbal memory tasks^14^, as well as attentional, goal directed and cognitive control processes^23^,^26^,^27^. Indeed, both the bilingual and IA participants, but not the French monolinguals, strongly recruited right middle frontal gyrus/posterior superior frontal gyrus (BA 6) and left medial frontal cortex in regions that precisely matched those observed in a meta-analysis that implicated these regions in non-verbal, as opposed to verbal, memory processing^14^. In contrast, the monolingual speakers activated bilateral middle/superior frontal gyrus (BA 9), which is commonly implicated in verbal memory processing^21^. Moreover, the bilingual and IA participants both activated bilateral superior temporal gyrus (STG; right >left) more strongly than the French monolinguals. Prior research has demonstrated greater bilateral activation during language tasks^37^, particularly in frontal, temporal and parietal regions^38^ for bilinguals who acquired their second language early in life, consistent with the experience of our bilingual group. Furthermore, right hemisphere STG activation has typically been associated with the processing of music and non-language sounds, in contrast to fine-grained phonetic discriminations of the kind required in the present study^39^,^40^.

Greater bilateral activation of several brain regions, as well as additional recruitment of attention and cognitive control regions, has previously been shown in bilingual speakers who are thought to use executive and cognitive control functions during the online use of two languages^26^. However, it is noteworthy in the present study that a similar pattern was also observed in the IA participants whose only exposure to another language had been discontinued years earlier. The activation of these regions by both the IA and bilingual groups, who performed this PWM task with high speed and accuracy, suggests that they drew on alternative systems to attain the same level of performance as the monolinguals. This interpretation is supported by previous studies in which structural changes in frontal, temporal and parietal regions involved in cognitive control^41^, as well as greater connectedness and efficiency in subnetworks involved in language monitoring^42^, have been observed in bilinguals in comparison to monolinguals, particularly as proficiency increases^41^. In the present study, more exposure to French was associated with greater activation in frontal and temporal/parietal brain areas that are comparable to those shown in these studies, suggesting that engagement of these executive and cognitive control regions may allow for increasingly efficient and proficient processing of a second language overtime^43^. This speaks to the remarkable flexibility of the brain to adapt to changing environmental circumstances and to engage alternative neural systems in new learning if other systems are not as readily available or relevant. Moreover, the similarities observed between IA and bilingual participants' brain activation patterns imply that even relatively early language experiences influence the way this system is established and subsequently used. These similarities may also suggest a relationship between early language experience and the development of executive function, particularly as it relates to cognitive advantages that have been observed in bilinguals^28^. This would be an interesting area for future research. Given that our participants were children and adolescents (ranging in age from 10 to 17 years at the time of testing), we were not able to determine whether or how this impacted their earlier development, when they were first acquiring French, nor whether these effects extend past adolescence into adulthood.

We assume that the neural processing differences observed between the present groups were due to the early establishment of neural representations for a language other than French (that is, Chinese). Evidence that early-established neural representations are, in fact, maintained over time comes not only from the present study, but also from a prior investigation of the same IA participants that examined the neural maintenance of their discontinued first language—Chinese^5^. Results from that study showed that the IA participants, who had early but discontinued exposure to Chinese, recruited the same brain regions when processing Chinese lexical tone as Chinese–French bilingual speakers who had spoken Chinese since birth. Crucially, even though the IA participants had been exposed to and used French exclusively since adoption, they showed activation that differed from the French monolinguals. Together with the current findings, this provides strong evidence that neural representations acquired during the earliest stages of development are maintained across time even in the absence of continued exposure to the source of that information. The former results^5^ demonstrate the maintenance of neural templates even when a language is discontinued, while the present study extends this finding to suggest that these templates have an ongoing and lasting impact on the processing of subsequently learned language sounds. While we have examined a tonal/non-tonal language pairing, it would also be interesting to assess the effect of early language experience on subsequent neural outcomes in other language pairs, perhaps as a function of language similarity. Indeed, there is some evidence that L2 processing might be more likely to recruit distinct brain areas if the L2 is dissimilar to the L1 (for example, Chinese versus English^44^). Thus, it would be interesting to examine processing of an L2 that is relatively similar to the L1 of participants, such as Spanish and French, in contrast to the language pairs examined in the present study that are very different, to see if the same pattern of results is obtained.

Principles supporting our interpretation have been shown in other species. For example, rats that have previously learned a spatial navigation task^45^ or a fear-conditioning task^46^ acquire a second version of the task via fundamentally distinct molecular mechanisms. This is because memory traces underlying the first task require that the animals learn the second task in a different way, by building on these earlier traces. Rather than recruiting the mechanisms necessary for first-time learning, new mechanisms were recruited to update previously established traces with new representations. Similarly, our work suggests that if infants acquire a system of phonological representations for one language, the phonology of a second language may subsequently be acquired or processed via distinct mechanisms that build on this early system. If this is the case, then the neural processing of French pseudo-words by the IA and bilingual participants in the present study might thus be argued to reflect the processing of new information within a system based on previously acquired neural representations that have been maintained overtime. Note that this does not imply that second-language learners will not recruit the same brain regions as native speakers when processing any aspect of their second language. Indeed, it has been demonstrated that certain neural patterns come to resemble those of native speakers as second-language learners' proficiency increases^47^. However, the results from the present study suggest that proficiency may not be the whole story; thus, beginning to elucidate the dynamic interaction between proficiency, the timing of language experience, and the neural representations of language and language sounds^48^.

In summary, the present study provides neural evidence that very early language experiences have a lasting influence on the way the brain processes the sounds of a language. We suggest that this is due to representations established from input in the first language that have persisted over time to influence the processing of second-language phonology. The fact that these neural differences did not preclude the achievement of equally advanced language proficiency highlights the incredibly adaptable ways that the brain is able to respond to a variety of language-learning circumstances.

## Methods

### Participants

Participants are the same as those reported by Pierce *et al.*^5^ Participants were right-handed and had no known hearing or neurological issues. Three groups participated (*n*=43): (1) IA participants adopted into French-speaking families before age three, who spoke only French at the time of the study (*n*=21; mean age: 13.7 years, range: 10;4–17;2; mean age at adoption: 12.6 months, range: 6–25 months), (2) Chinese–French bilinguals who learned Chinese from birth, began acquiring French as a second language by age three and spoke both French and Chinese at the time of the study (*n*=12; mean age: 13.0, range: 9;10–16;6; mean age of French onset: 17 months, range: 0–36 months), and (3) French monolinguals who had never been exposed to Chinese (*n*=10 mean age: 13.5; range: 10;1–17;0). Consistent with the adoption demographics of China and to ensure comparability with the female IA participants, all participants were female^49^,^50^. Chinese adoptees typically do not show cognitive or socio-emotional deficits or disadvantages due to their pre-adoption experiences^50^,^51^,^52^, making them particularly suited to the purpose of the present study. This was confirmed within our sample through parent questionnaires and behavioural assessments see also (ref. 12). Oral and written informed consent was obtained from participants' caregivers and from each participant before beginning the experiment, which was approved by the research ethics board of the Montreal Neurological Institute. Sample sizes are comparable to those used in similar studies^23^. Of note, while we report data for the full groups of participants, we also conducted the same analyses on subgroups (*n*=10 per group) matched for age, and age of exposure to French in the case of bilingual and IA participants. Results from the analyses with full groups and subgroups were comparable, and thus we are reporting only the full groups here. Four additional participants were tested but their data were not analyzed. One IA participant was left-handed and one did not complete the full experimental session. One monolingual participant was left-handed and one had dental braces that caused extensive artefacts in the brain images.

### Stimuli

Stimuli consisted of 36 bisyllabic French pseudo-words (for example, *vapagne*, *chansette*) taken from Chee *et al.*^23^ For the present study, all stimuli were recorded by a female native speaker of French. Pseudo-words were chosen to examine responses to French phonology without reliance on learned lexical information. Pseudo-words were constructed to have a CVCVC (C, consonant; V, vowel) phonetic structure and differed from real French words by one phoneme. Lexical frequency and neighbourhood were not actively controlled; however, because our participants demonstrated comparable vocabulary and language scores (see behavioural analysis) it is unlikely that this would have affected the results. (A description of the original stimuli can also be found in ref. 53).

### Behavioural assessment

Participants' parents completed two questionnaires—the Questionnaire sur le developpement et l'acquisition du langage chez les enfants (Questionnaire on children's language development) and the Questionnaire: Exposition au francais et a chinois (Questionnaire: Exposure to French and Chinese)^52^. The first obtained information on participants' early developmental history, including families, socioeconomic status, language exposure, current cognitive, socio-emotional, and health status, and adoption history for the IA participants. The second questionnaire obtained detailed information about participants' language environments in several contexts (for example, home and school) from birth to the time of testing. Parents reported that French monolingual children heard and used exclusively French every day, and Chinese–French bilinguals heard and used both French and Chinese every day, except for one who spoke mostly French with occasional (but highly proficient) Chinese. All IA children were exposed to Chinese as their first language but heard and used only French since adoption, with the exception of three children who experienced brief exposure to Chinese (that is, through a one-time culture course, a visit to China and friends with Chinese parents). Results did not differ when these participants were excluded from the analyses. Detailed language background information of these participants is also reported by Pierce *et al*.^5^

Language and general cognitive measures were used to assess expressive vocabulary (Expressive One-Word Picture Vocabulary Test in French (EWOPVT)^54^), receptive vocabulary (Echelle de vocabulaire en images Peabody (EVIP)^55^), general verbal and PWM (Wechsler sentence repetition task in French and the French non-word repetition test^56^), and spatial memory (Wechsler block design subtest). Inclusion of the spatial memory test allowed us to ensure that differences between groups were language specific. Speech samples were also collected from all participants in the form of paragraphs that they read aloud, ensuring equal proficiency across groups.

### Brain imaging

Image acquisition was performed on a 3T Siemens Trio scanner at the Montreal Neurological Institute (MNI). A global anatomical three-dimensional T1-weighted, gradient-echo sequence (Magnetization Prepared Rapid Gradient Echo) scan was obtained for each participant, and motion correction was administered online to all functional sequences using three-dimensional Prospective Acquisition Correction^57^.

### BOLD functional magnetic resonance imaging

Participants heard French pseudo-words presented in succession and were instructed to respond with a button press when they heard the target word. Sounds were presented binaurally through foam insert headphones (Sensimetrics model S14 insert earphones) using a computer and E-Prime software (E-Prime 1.1, Psychology Software Tools). Stimuli were arranged in a block design of four blocks of three sets (one of each condition) presented in random order. Each block consisted of 12 consecutive pseudo-words. Pseudo-words were not used as targets in more than one set, and were not presented in more than one set within a block. Blocks began with a written and spoken prompt informing participants whether to respond to targets in the 0-back, 1-back, or 2-back positions. In the 0-back condition participants were required to respond every time they heard the first stimulus presented in the series. In the 1-back condition participants were required to respond each time a stimulus matched that presented in the prior position (that is, two identical stimuli in a row). In the 2-back condition participants were required to respond each time a stimulus matched that presented two positions prior. Each series contained four target stimuli. There were 30 s of silence between blocks during which participants viewed a central fixation cross. Experimenters were not blind to participants' group membership; however, testing was carried out via computer and experimenters and technicians were outside the scanner room, thus experimenter bias was not a factor.

Four series of 72 functional images were acquired with the following characteristics: gradient echo, TE=30 ms, TR=3 s, matrix size: 64 × 64, voxel size: 3.5 × 3.5 × 3.5 mm^3^. Images were acquired following the presentation of each pseudo-word. Accuracy and reaction time (measured from the onset of the target word) were collected to measure task performance. Before scanning participants were trained on the task until reaching at least 80% accuracy to ensure that any differences observed could not be attributed to differences in performance. Complete accuracy and reaction time scores were not available for one monolingual, one bilingual and five IA participants owing to a software error; however, these participants' all exhibited the same high accuracy before testing, during the training period.

### fMRI analysis

FSL software was used to perform statistical analyses on these functional data^58^. fMRI data were processed using FEAT (ref. 59). Preprocessing included spatial smoothing with a 6-mm-full width at half maximum (FWHM) Gaussian filter, slice-timing correction and high-pass temporal filtering. The design matrix of the linear model was convolved with a hemodynamic response function modelled as a difference of two gamma functions timed to coincide with the acquisition of each slice. Data from each run was registered to participants' own t1-weighted anatomical image, which was brain extracted using BET (ref. 60), and then normalized to the MNI template (MNI 305) (ref. 61). Individual runs within participants were combined using a fixed-effects analysis. Within-groups averages were obtained for each group using a mixed-effects linear model. Comparisons were conducted using a 3 × 3 ANOVA with the factors group (French monolingual, Chinese–French bilingual, IA) and condition (0-back, 1-back, 2-back), and included age and duration of exposure to French as covariates. To examine the effect of memory load, 2-back minus 0-back subtractions were additionally performed for each group. T-statistic images were thresholded using a cluster threshold of 2.3, corrected for multiple comparisons. Threshold significance was established as *t*=5.17 for the whole-brain activation peaks^62^. Anatomical locations were determined using the Talairach client software (version 2.4.3).

To determine whether bold signal changes were modulated by individual differences in language experience, whole-brain, voxel-wise linear regressions were performed within 2-back minus baseline and 2-back minus 0-back subtractions using the fMRIstat software. Covariates for each regression analysis were age of acquisition onset of French (AoA) and length of time exposed to French. Because *a priori* predictions about location of activation were made, a cluster-based threshold of 4.3 was applied.

To determine whether each group recruited similar or distinct networks of activation when processing French phonological units, a PPI analysis was applied using the FSL software^30^,^58^. PPI analysis measures task-related functional connectivity by measuring the correlation between a seed region and other brain regions across time to determine whether the correlation in activation changes depending on the task being performed. The seed region was defined as an 8 mm radius around the left insular peak from each group. A mask of this region was created for each group in standard space (MNI 152 2 mm) and this was registered to the functional space of each participant using non-linear registration. The mean time series within this region was extracted from each participant and each condition. A model was then created to examine the interaction term between the time series and the 2-back task. Results specify regions whose correlation with left insula is dependent on whether the task is being performed^30^. A significance threshold of *t*=3.17 was applied as is typical for this type of analysis^30^.

## Additional information

**How to cite this article:** Pierce, L. J. *et al.* Past experience shapes ongoing neural patterns for language. *Nat. Commun.* 6:10073 doi: 10.1038/ncomms10073 (2015).

## Figures and Tables

**Table 1:** Coordinates of maximum peaks within significant clusters for 3 × 3 ANOVA main effects of group (French monolingual, Chinese–French bilingual, international adoptee) and condition (0-back, 1-back, 2-back), controlling for age and duration of exposure to French.

**Hemisphere**	**Region**	**BA**	**Group**				**Condition**			
			***x***	***y***	***z***	***t***	***x***	***y***	***z***	***t***
Left	Superior frontal gyrus	8					−4	16	50	5.27
	Inferior frontal gyrus	45	−38	28	6	7.01				
	Anterior insula	13	−30	22	−10	5.39				
	Superior temporal gyrus	38	−54	8	−14	5.50				
	Superior temporal gyrus	39	−44	−50	26	6.03				
	Inferior temporal gyrus	20	−60	−18	−16	5.63				
	Inferior parietal lobule	40	−40	−44	52	5.83	−38	−46	46	5.42
	Posterior cingulate	31	−18	−54	26	6.19	−8	−54	22	5.63
	Posterior cingulate	23					−6	−54	16	5.19
Right	Frontal operculum/anterior insula	13					34	20	0	5.52
	Anterior cingulate	25					4	18	−8	5.82
	Middle frontal gyrus	6					28	4	56	6.44
	Precentral gyrus	6	40	−8	48	5.89				
	Superior temporal gyrus	22	56	6	−2	6.35				
	Middle temporal gyrus	21	52	−36	−2	6.50				
	Superior parietal lobule	7	34	−46	48	5.39				
	Precuneus	7	12	−66	36	5.56				

ANOVA, analysis of variances; BA, Brodmann area; MNI, Montréal Neurological Institute.

Threshold *t*=5.17; *x*, *y* and *z* coordinates based MNI 305 template^61^.

**Table 2:** Coordinates of maximum peaks within significant clusters for between-group subtractions of monolinguals, bilinguals, and international adoptees, collapsed across conditions.

**Hemisphere**	**Region**	**BA**	**Monolingual>Bilingual**				**Monolingual>International Adoptee**				**Bilingual >Monolingual**				**International Adoptee >Monolingual**				**Bilingual >International Adoptee**				**International Adoptee >Bilingual**			
			***x***	***y***	***z***	***t***	***x***	***y***	***z***	***t***	***x***	***y***	***z***	***t***	***x***	***y***	***z***	***t***	***x***	***y***	***z***	***t***	***x***	***y***	***z***	***t***
Left	Frontal pole	10					−48	46	−4	5.38																
	Frontal operculum/anterior insula	45	−38	28	6	7.39	−38	28	6	5.37																
	Inferior frontal gyrus	47	−30	22	−10	5.70																				
	Inferior temporal gyrus	20													−62	−16	−16	5.71								
	Superior temporal gyrus	39									−44	−50	26	6.39												
	Inferior parietal lobule	40					−40	−44	52	6.24																
	Supramarginal gyrus	40													−44	−50	32	6.10								
	Posterior cingulate	31									−18	−54	26	6.20	−18	−56	26	6.12								
	Lingual gyurs	18													−8	−84	−2	5.32								
Right	Frontal operculum/anterior insula	45	40	22	14	5.33																				
	Middle frontal gyrus	9	50	22	30	5.20																				
	Precentral gyrus	6													40	−10	42	6.03								
	Superior temporal gyrus	22									56	8	−2	5.71	56	6	−2	6.63								
		22									48	−6	2	5.53												
	Middle temporal gyrus	21	52	−36	−2	6.34	52	−36	−4	6.62																
		21	46	−42	14	6.46																				
		21					62	−38	−4	5.30																
	Superior parietal lobule	7					34	−46	48	5.63													46	−62	52	5.25
	Precuneus	7									12	−66	36	5.63	12	−68	34	5.53								

BA, Brodmann area; MNI, Montréal Neurological Institute.

Threshold *t*=5.17; *x*, *y* and *z* coordinates based MNI 305 template^61^.

**Table 3:** Coordinates of maximum peaks within significant clusters for comparisons of the 0-back, 1-back, and 2-back conditions collapsed across groups.

**Hemisphere**	**Region**	**BA**	**2-back >0-back**				**2-back >1-back**				**1-back >0-back**				**1-back >2-back**				**0-back >1-back**				**0-back >2-back**			
			***x***	***y***	***z***	***t***	***x***	***y***	***z***	***t***	***x***	***y***	***z***	***t***	***x***	***y***	***z***	***t***	***x***	***y***	***z***	***t***	***x***	***y***	***z***	***t***
Left	Superior frontal gyrus	8	−4	16	50	5.19																				
	Inferior parietal lobule	40	−38	−46	46	5.79																				
Right	Anterior insula	13	32	20	6	5.30	34	20	0	5.48																
	Subcallosal gyrus	25													4	18	−8	5.28					−6	14	−10	5.89
	Middle frontal gyrus	6	28	4	56	6.53	28	4	56	5.42																
	Posterior cingulate	31																					−8	−54	22	5.84

BA, Brodmann area; MNI, Montréal Neurological Institute.

Threshold *t*=5.17; *x*, *y* and *z* coordinates based MNI 305 template^61^.

**Table 4:** Coordinates of peak activation for groups of monolinguals, bilinguals, and international adoptees, in 0-back, 1-back, and 2-back conditions.

**Hemisphere**	**Region**	**BA**		**Monolingual**				**Bilingual**				**International adoptee**			
				***x***	***y***	***z***	***t***	***x***	***y***	***z***	***t***	***x***	***y***	***z***	***t***
Left	Superior frontal gyrus	10	2-back									−32	54	16	7.59
		8	0-back	−6	10	60	6.29					−2	14	50	6.20
		8	2-back	−2	18	50	7.08	−2	16	54	8.14				
	Inferior frontal gyrus/anterior insula	13/45	0-back	−36	28	6	7.61					−34	20	2	6.32
		13/45	1-back	−32	22	2	6.83					−32	22	4	6.49
		13/45	2-back	−32	22	2	8.12	−32	22	4	7.95	−32	22	2	10.1
	Middle frontal gyrus	9	0-back	−42	22	30	5.36								
		9	2-back	−42	22	30	6.41					−40	26	30	6.88
		6	2-back					−28	4	58	5.19	−30	2	56	6.66
	Anterior cingulate	32	2-back					−12	18	38	5.21	−2	14	22	5.39
	Inferior frontal gyrus	9	0-back	−54	16	24	5.30								
	Superior temporal gyrus	38	0-back	−44	6	−16	5.68					−46	14	−8	5.64
		38	2-back	−44	6	−14	5.23								
		42	0-back	−60	−30	10	7.75	−62	−28	10	5.89	−60	−18	8	7.55
		42	1-back	−60	−30	10	6.68	−62	−26	10	5.59	−60	−16	8	6.59
		42	2-back	−62	−30	10	5.48	−64	−28	8	6.01	−58	−22	2	7.02
	Inferior temporal gyrus	20	2-back									−62	−30	−16	6.70
	Inferior parietal lobule	40	0-back									−50	−54	52	5.23
		40	1-back									−34	−50	40	5.80
		40	2-back	−40	−44	50	6.23	−34	−48	40	6.71	−34	−50	40	9.43
	Cerebellum		2-back					−26	−64	−30	5.51	−28	−60	−32	6.85
Right	Superior frontal gyrus	10	0-back									36	58	16	5.40
		10	2-back					34	58	12	6.33				
		8	0-back	0	18	52	5.58								
		8	1-back	2	18	52	5.28	8	20	50	5.35	4	20	50	6.63
	Medial frontal gyrus	8	0-back	4	38	36	5.23								
	Middle frontal gyrus	9	0-back	50	22	30	5.77								
		9	1-back									46	30	34	5.82
		9	2-back	48	32	36	7.48	48	32	34	6.64				
		6	2-back					28	14	60	6.55	30	10	58	7.20
	Anterior insula	13	0-back	34	22	4	7.19								
		13	1-back	32	24	−2	6.23	34	22	8	5.91	32	22	6	7.89
		13	2-back	32	22	0	7.95	34	22	6	9.25	32	22	6	11.00
	Anterior cingulate	24	2-back									4	16	22	5.63
	Thalamus		2-back									4	−6	18	7.15
	Superior temporal gyrus	38	1-back									46	6	−8	5.53
		22	0-back	56	−16	4	7.44	62	−12	2	7.31	66	−20	8	8.90
		22	1-back	60	−12	2	6.35	62	−12	4	7.12	66	−22	10	7.88
		22	2-back					62	−24	4	7.35	64	−22	8	8.87
		41	1-back	48	−40	12	5.56								
	Middle temporal gyrus	21	1-back	52	−36	−2	6.24					62	−34	−14	5.53
		21	2-back	54	−36	−2	6.44	56	−30	−10	5.43				
	Caudate		0-back					24	−40	20	5.64				
	Inferior parietal lobule	40	0-back	42	−50	52	5.76					50	−50	50	5.20
		40	1-back	42	−50	52	5.84					40	−50	46	7.24
		40	2-back	38	−50	46	6.86	38	−50	46	7.92	42	−50	44	9.80
	Cerebellum		2-back									28	−62	−28	5.52

BA, Brodmann area; MNI, Montréal Neurological Institute.

Threshold *t*=5.17; *x*, *y* and *z* coordinates based MNI 305 template^61^.

**Table 5:** Coordinates of peak activation for groups of bilinguals and international adoptees in a subtraction of 2-back minus 0-back conditions, indicating an effect of increased cognitive load.

**Hemisphere**	**Region**	**BA**	**Bilingual**				**International adoptee**			
			***x***	***y***	***z***	***t***	***x***	***y***	***z***	***t***
Left	Inferior parietal lobule	40					−34	−50	42	5.84
Right	Middle frontal gyrus	6	28	16	48	5.22	38	32	36	5.37
		6	30	8	56	5.23	36	30	30	5.24
	Globus pallidus						16	4	0	5.19
	caudate						18	−4	22	5.19
	Inferior parietal lobule	40	38	−42	48	5.29	40	−46	48	5.80

BA, Brodmann area; MNI, Montréal Neurological Institute.

Threshold *t*=5.17; *x*, *y* and *z* coordinates based MNI 305 template^61^.

**Table 6:** Coordinates of peak activation for PPI analysis of monolinguals, bilinguals, and international adoptees.

**Hemisphere**	**Region**	**BA**	**Monolingual**				**Bilingual**				**International Adoptee**			
			***x***	***y***	***z***	***t***	***x***	***y***	***z***	***t***	***x***	***y***	***z***	***t***
Left	Middle temporal gyrus	21	−64	−20	−14	3.44								
	Postcentral gyrus	1	−52	−20	48	3.30								
		3	−28	−22	48	4.42								
	Precentral gyrus	4	−32	−24	66	3.87								
	Supramarginal gyrus	40	−58	−46	44	3.51								
	Inferior parietal lobule	40	−48	−48	38	3.29								
	Angular gyrus	39	−50	−66	34	4.18								
Right	Frontal pole	10	0	64	20	3.82					6	64	16	4.06
	Middle frontal gyrus	11									44	44	−10	3.64

IA, internationally adopted; MNI, Montréal Neurological Institute; PPI, psychophysiological interactions; PWM, phonological working memory.

Coordinates represent regions that are functionally connected to left anterior insula during task performance. Note that for monolinguals the insula is functionally connected to several regions implicated in the PWM network, while this is not the case for bilinguals or IAs.

Threshold *t*=3.17; *x*, *y* and *z* coordinates based MNI 305 template^61^.

**Table 7:** Coordinates of peak activation from a whole brain voxel-wise regression for bilinguals and international adoptees (*n*=32) showing regions where amount of exposure to French is positively associated with blood oxygenation level dependent (BOLD) activation.

**Hemisphere**	**Region**	**Exposure to French 2-back—baseline**				**Exposure to French 2-back—0-back**			
		***x***	***y***	***z***	***t***	***x***	***y***	***z***	***t***
Left	Superior frontal gyrus	−2	22	50	4.52				
	Caudate	−18	0	22	4.66				
	Supramarginal gyrus	−32	−48	36	5.30				
	Angular gyrus	−44	−56	54	5.69				
	Lateral occipital cortex	−40	−60	60	4.71				
	Cerebellum	−54	−60	−28	4.44				
Right	Middle frontal gyrus	32	0	70	4.65	38	30	34	5.76
		34	28	28	4.58	36	6	38	4.51
	Frontal orbital cortex	36	28	0	4.71				
	Frontal pole	36	58	16	5.47				
	Caudate					12	−4	16	5.30
	Middle temporal gyrus	60	−20	−10	4.50				
	Angular gyrus	44	−52	48	4.41	44	−46	50	4.50

MNI, Montréal Neurological Institute.

Threshold *t*=4.3; *x*, *y* and *z* coordinates based MNI 305 template^61^.
